# Volatile Compounds Emitted by *Pseudomonas aeruginosa* Stimulate Growth of the Fungal Pathogen *Aspergillus fumigatus*

**DOI:** 10.1128/mBio.00219-16

**Published:** 2016-03-15

**Authors:** Benoit Briard, Christoph Heddergott, Jean-Paul Latgé

**Affiliations:** Aspergillus Unit, Pasteur Institute, Paris, France

## Abstract

Chronic lung infections with opportunistic bacterial and fungal pathogens are a major cause of morbidity and mortality especially in patients with cystic fibrosis. *Pseudomonas aeruginosa* is the most frequently colonizing bacterium in these patients, and it is often found in association with the filamentous fungus *Aspergillus fumigatus*. *P. aeruginosa* is known to inhibit the growth of *A. fumigatus* in situations of direct contact, suggesting the existence of interspecies communication that may influence disease outcome. Our study shows that the lung pathogens *P. aeruginosa* and *A. fumigatus* can interact at a distance via volatile-mediated communication and expands our understanding of interspecific signaling in microbial communities.

## IMPORTANCE

Microbiota studies have shown that pathogens cannot be studied individually anymore and that the establishment and progression of a specific disease are due not to a single microbial species but are the result of the activity of many species living together. To date, the interaction between members of the human microbiota has been analyzed in situations of direct contact or liquid-mediated contact between organisms. This study showed unexpectedly that human opportunistic pathogens can interact at a distance after sensing volatiles emitted by another microbial species. This finding will open a new research avenue for the understanding of microbial communities.

## OBSERVATION

The filamentous fungus *Aspergillus fumigatus* and the bacterium *Pseudomonas aeruginosa* occupy similar environmental niches. In addition, both of these organisms are potent opportunistic pathogens, frequently coexisting in the human lung during colonization of mucous deposits that accumulate in the airways of patients with cystic fibrosis (CF) or invasive infections among immunocompromised patients. This shared habitat in both nature and human infection suggests the existence of competitive interactions between the two species that could influence microbial pathogenicity and disease outcome. For example, it is known that when *P*. *aeruginosa* is in direct contact with *A*. *fumigatus*, *P*. *aeruginosa* releases toxic molecules that inhibit the growth of the fungus (1, 2). Emerging studies have shown that communication between microbial species involves not only water-soluble compounds but also the release and detection of volatile organic compounds (VOCs). The importance of VOCs in ecology has been underestimated for a long time, but recent and ongoing work emphasizes the role of microbial communication via the gas phase (3, 4). VOCs have been repeatedly reported to inhibit fungal growth. For example, *Pseudomonas* subsp. and *Burkholderia* subsp.-derived VOCs have inhibitory effects on *Rhizoctonia*, *Alternaria*, or *Fusarium* species (5–7). *Aspergillus fumigatus* was inhibited by volatile antimicrobials from an endophytic fungus (8). Here we demonstrate the occurrence of an unexpected stimulatory effect on the growth of *A*. *fumigatus* by *P*. *aeruginosa* which is promoted at a distance without direct contact between the two species. The effect is mediated via the gas phase, and the volatile compound responsible for this effect is dimethyl sulfide. This finding establishes a new paradigm in the understanding of interactions between members of the microbiota, which has important implications for both the initiation and progression of coinfections by these two pathogens.

**FIG 1:**
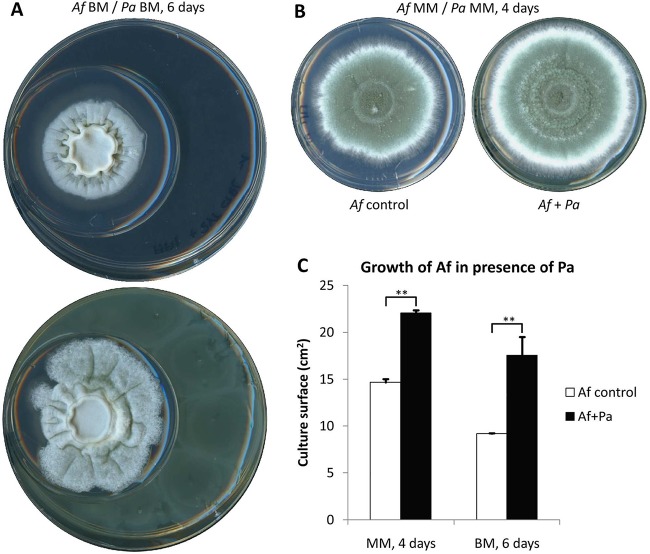
Growth characteristics of *A*. *fumigatus* in a plate-in-plate (PIP) coculture assay. (A) The *A*. *fumigatus* (*Af*) fungus is isolated from a separate compartment containing either medium alone (top) or medium inoculated with *P*. *aeruginosa* (*Pa*) (bottom). The presence of *P*. *aeruginosa* increases mycelial growth, particularly in the vicinity of the bacterium. The medium used was Brian medium (BM). (B) The growth-promoting effects of *P*. *aeruginosa* on *A*. *fumigatus* were also evident on minimal medium (MM), although asymmetric growth of the colony was not observed. (C) Quantitation of the surface area of *A*. *fumigatus* colonies growing on MM or BM in sealed PIP cultures in the presence and absence of *P*. *aeruginosa*. Values are averages plus standard errors of the means (SEM) (error bars) of at least three replicates. Values that are significantly different (*P* ≤ 0.01) are indicated by a bar and two asterisks.

**FIG 2:**
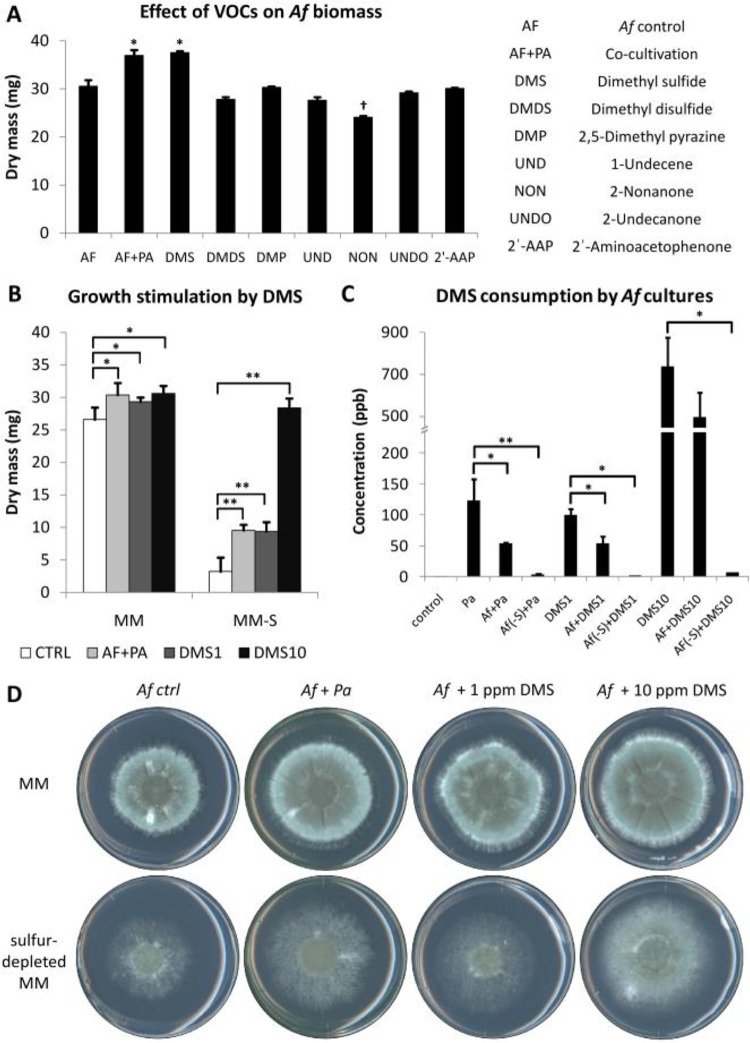
Effects of VOCs on the growth of *A*. *fumigatus*. (A) Quantitation of *A*. *fumigatus* (*Af*) biomass in the presence of 100 ppm of pure VOCs identified in the *P*. *aeruginosa* culture headspace. Only DMS significantly increased growth, and only 2-nonanone had a significant inhibitory effect. (B) Comparison of the growth-promoting effects of 1 ppm DMS (DMS1) and 10 ppm DMS (DMS10) on sulfur-replete MM and on sulfur-deficient MM (MM-S). CTRL, control. (C) Residual concentration of DMS in the culture headspace of the double-plate setup. DMS produced by *P*. *aeruginosa* (grown on MM) or the pure compound (1 and 10 ppm) is partially consumed by the *A*. *fumigatus* fungus when grown on MM (*Af*) but is fully consumed on sulfur-deficient MM [*Af*(-S)]. (D) Agar plates showing the growth stimulation of *A*. *fumigatus* by DMS. As shown in panel B, the effect is increased when the fungus is grown under sulfur starvation. All error bars show the SEM of the mean values. Values that are significantly different are indicated as follows: *, *P* ≤ 0.05; †, *P* ≤ 0.05; **, *P* ≤ 0.01.

Figure 1A illustrates the plate-in-plate (PIP) method that was used to determine how *P*. *aeruginosa*-derived volatiles affect the growth of *A*. *fumigatus* without direct contact between the two organisms. *A*. *fumigatus* conidia were inoculated into the center of a small plate that was positioned asymmetrically within a larger petri plate containing medium inoculated with *P*. *aeruginosa*. The two plates were sealed, and radial growth of the fungus was monitored over time. In this assay, VOCs released by the two organisms are confined in the shared headspace. Two defined media were used: minimal medium (MM) (9), which supports rapid radial growth, and Brian medium (BM) (10), which supports slower growth. *P*. *aeruginosa* fully covered the surface of its compartment within 2 days after inoculation in both media. Surprisingly, the presence of *P*. *aeruginosa* in the adjacent compartment led to a stimulation of fungal growth on both types of medium (Fig. 1). On BM, an asymmetric augmentation of colonial growth at the colony boundary that was closest to the highest concentration of bacteria suggests the presence of a diffusion gradient of bacterium-derived VOCs that stimulates invasive outgrowth of the fungus (Fig. 1A). Since only volatiles are able to pass the barrier between the plates in this assay, the volatome produced by the two organisms in single cultures and cocultures was analyzed using solid-phase microextraction (SPME) and gas chromatography-mass spectrometry (GC-MS), as described previously (11). The VOCs produced by *P*. *aeruginosa* under these culture conditions were identified as dimethyl sulfide (DMS), dimethyl disulfide (DMDS), 2,5-dimethylpyrazine (2,5-DMP), 1-undecene, 2-nonanone, 2-undecanone, and 2′-aminoacetophenone (2′-AAP), consistent with what has previously been described for this bacterium (12). To identify which volatile was responsible for the growth-promoting effects on *A*. *fumigatus*, we tested pure bacterial VOCs with concentrations in the range of 10 to 100 ppm (Fig. 2A). DMS was the only VOC tested that augmented the growth of *A*. *fumigatus*. The compound was stimulatory at very low concentrations (1 ppm [Fig. 2B]), which corresponded to the amount released by the bacterium grown alone for 3 days (Fig. 2C). Since DMS contains sulfur, the possibility that it could serve as a nutrient source was tested on sulfur-depleted medium (specific formulation described in “Strains and culture conditions” below). *A*. *fumigatus* grew poorly under these conditions, both in terms of radial outgrowth and total biomass. DMS could rescue these growth defects, and it was completely resorbed by the fungus, suggesting that its growth stimulatory activity involved nutrient sensing and S-compound uptake (Fig. 2B to D). The related sulfur-containing volatile DMDS could also stimulate *A*. *fumigatus* growth under sulfur starvation conditions, but it had no growth stimulatory effects in S-replete medium (see Fig. S1 in the supplemental material). These findings were consistent with the observation that organic S compounds are essential for the growth of *A. fumigatus* (13). DMS stimulated *A*. *fumigatus* growth even when up to 8 mM of inorganic sulfate was present in the minimal medium in which *A*. *fumigatus* produces only traces of DMS and DMDS. In contrast, the effect of *P*. *aeruginosa* DMS was not seen when the fungus was grown in the presence of nonphysiological free concentrations of organic sulfur such as 5 mM methionine in an MM medium or in a peptone-based medium (Sabouraud) which contains an equivalent of 2.5 mM methionine, because in these media *A*. *fumigatus* itself released >5 times the amount of DMS produced by *P*. *aeruginosa* (data not shown). Other Gram-negative bacteria such as *Escherichia coli* and *Burkholderia cepacia* are able to stimulate the growth of *A*. *fumigatus*, showing that the production of stimulatory volatiles is not restricted to *P*. *aeruginosa* (data not shown). These results indicate that under sulfur starvation, *A*. *fumigatus* could use exogenous volatile bacterial organic compounds to promote vegetative growth *in vivo*.

It is well established that microbes that exist in complex multicellular consortia secrete chemical shields that restrict the growth of competing species. To date, the study of these interspecies interactions in microbial communities has been largely focused on the effects of direct contact or metabolic exchange with water-soluble molecules, the effects of which are entirely inhibitory (14–17). We show here that these interactions can also occur at a distance, without any direct physical contact between organisms. Moreover, we report for the first time that the volatiles released by *P*. *aeruginosa* have the surprising effect of being stimulatory to the growth of *A*. *fumigatus*. This finding contrasts with the findings of previous studies in plant fungal pathogens, where the volatiles released by *Pseudomonas* and *Burkholderia* species have inhibitory effects on *Rhizoctonia solani*, *Alternaria alternata*, and *Fusarium proliferatum* (5–7). Based on analysis of the interactions between *P*. *aeruginosa* and *A*. *fumigatus* (2; this study), microbial invasion of the lung can be seen as a two-step event. When *P*. *aeruginosa* and *A*. *fumigatus* are separated, volatiles released by *P*. *aeruginosa* favor the invasion of the lung parenchyma by *A*. *fumigatus*, but as soon as the two microorganisms have colonized the lung and are in direct contact, the mutualistic interaction that supported the substrate invasion becomes antagonistic when each microbe competes for its own nutrients. Since the VOCs produced by *P*. *aeruginosa in vitro* have also been detected in sputum samples from CF patients (18), our findings raise the possibility that infection of the CF lung by *P*. *aeruginosa* and the release of growth-promoting VOCs may predispose to *A*. *fumigatus* cocolonization by creating an environment that is more conducive to the germination of inhaled *A*. *fumigatus* conidia or to increase fungal burden during coinfection and thus contribute to the cycles of acute exacerbation that are frequently associated with CF. This would be in accordance with clinical data showing a positive correlation between coinfection with *A*. *fumigatus* and *P*. *aeruginosa* and a more rapid decline in lung functionality (19, 20). Methylated sulfur compounds are oxidation products of methanethiol, but the pathway responsible for the production of DMS in *P*. *aeruginosa* is not known. In *Pseudomonas putida*, methanethiol is generated by a methionine lyase. However, *P*. *aeruginosa* does not possess this enzyme, the most similar being a cystathionine lyase. In *Lactobacillus helveticus*, this enzyme is able to use methionine to produce S-VOCs. However, a null mutant of the cystathionine lyase still produced the S-VOCs, which suggested that other enzymes and/or even a nonenzymatic cleavage of methionine in the presence of pyridoxal-5′-phosphate may be responsible for the production of DMS (21). In conclusion, our findings add a new level of complexity to the understanding of how microbes interact and suggest that these volatile-mediated effects may lead to alterations in the balance of microbial populations in the CF lung that impact disease pathogenesis.

### Strains and culture conditions.

*Aspergillus fumigatus* CBS144-89 was grown on 2% (wt/vol) malt− 2% (wt/vol) agar slants for 1 week, and conidia were harvested in water containing 0.05% (vol/vol) Tween 20. *Pseudomonas aeruginosa* PAO1 was grown in 2× YT medium (16 g/liter tryptone, 10 g/liter yeast extract, 5 g/liter NaCl, pH 7.0) and harvested by centrifugation, washed with phosphate-buffered saline (PBS), and immediately used for inoculation of the PIP agar plates. For these experiments, the organisms were grown on modified minimal medium (6) with 20 mM glutamine instead of ammonium tartrate as the nitrogen source or on Brian’s broth (7). In both media, sulfur is present as sulfate at a concentration of 2.1 mM (minimal medium [MM]) or 8.1 mM (Brian medium [BM]), respectively. Minimal medium without sulfur was prepared by replacing all sulfates with their corresponding chlorides, while keeping the molarity of the respective trace metal. The media were prepared as 2-fold stock solutions and sterilely filtered (Steritop; Millipore). This stock was prewarmed to 65°C and mixed with an equal volume of liquid 1.6% (wt/vol) agarose (Life Technologies) in water at the same temperature.

The experimental device used for the PIP experiments is shown in Fig. S2A in the supplemental material. The lid of a small round 55-mm-diameter petri dish was inverted and placed asymmetrically within a 90-mm plate. Eight milliliters of medium was added to the inner small plate, and 11 ml of medium was added to the outer plate. The total headspace within the large plate amounts to 50 cm^3^. A 5-µl suspension of *A*. *fumigatus* conidia at 2 × 10^7^/ml^−1^ was placed in the center of the inner plate. For the outer plate, 100 µl of a freshly prepared *P. aeruginosa* suspension at an optical density of 0.1 was streaked onto the agar. The plates were immediately wrapped with 2 layers of Parafilm or sealed into a 50-µm polypropylene bag (12.5 by 15 cm). The effects of pure volatile organic compounds (VOCs) (Sigma-Aldrich) were tested by spotting up to 100 ppm onto a filter paper contained within a 5-mm plastic vessel and placing the vessel on the agar surface. For concentrations inferior to 10 ppm (0.5 µl), VOCs were diluted in water (1% [vol/vol] dimethyl sulfide [DMS], 0.2% [vol/vol] dimethyl disulfide [DMDS]).

### Growth measurement.

Growth was estimated either by the diameter of the colony in two perpendicular directions or by the dry weight of the colony. For the dry weight determination, the plate was placed for 40 s in an 800-W microwave to melt the agar; the colony was washed extensively with 65°C water and dried for 48 h at 70°C before weighing.

### Volatile measurement by SPME.

Solid-phase microextraction (SPME) analysis was carried out as previously described (8), with the following experimental modification. The SPME fiber was inserted directly into the petri dish through a small hole drilled just before the measurement. After 30 min, the volatiles were identified by GC-MS after subtracting the background originating from the plastic ware. Because of coelution of other hydrocarbons at retention times similar to those of the sulfur VOCs (e.g., DMS and ethanol), typical fragment ions were selected as a quantification base. An *m*/*z* = 62 was used for DMS, and an *m*/*z* of 94 was used for DMDS. Both ions had no background at all at the retention times of these VOCs (1.03 min for DMS and 3.42 min for DMDS at a column temperature of 30°C). The peaks of the selected ions were manually integrated using the methods described above.

Measurement of the concentration of volatile compounds produced by a growing aerobic organism is complicated by the fact that an air-tight seal creates a hypoxic environment that restricts growth, whereas an open system constantly loses VOCs. For this reason, bags that completely prevent the loss of volatiles (GENbags; bioMérieux) were inadequate for this study because of limited fungal and bacterial growth (data not shown). Embedding the plates in Parafilm prevented hypoxia, but the VOC loss was approximately 1.5-fold higher than sealing the plates into 50-µm polypropylene bags. Using the bags that permit limited gas exchange was thus an effective compromise to balance fungal growth with VOC containment.

For the sulfur-containing VOCs, we performed a calibration to be able to correlate GC-MS data (ion reads) to absolute VOC concentrations in the headspace. Since the early retention times of DMS and DMDS are within the injection peak of carrier solvents like methanol, direct standard injection is not possible. To circumvent this problem, we prepared a plate and sealed bag setup and inoculated known amounts of DMS and DMDS on the filter of the test setup. After 1, 2, and 3 days of incubation, the amounts of VOCs extractable by SPME were measured. For both DMS and DMDS, the loss rate is about 50% per day. This experiment enabled us to quantify the absolute VOC concentration (see Fig. S2B in the supplemental material). On MM, large amounts of DMS and DMDS were produced between 1 and 3 days. This indicates that the evaluation of the impact of the bacterial volatiles on fungal growth on MM is best after 3 days. In Brian medium, the production of VOCs gradually increased over time for the 7-day experimental period. This would explain why the effect of the *P*. *aeruginosa* volatiles is best seen after 6 days of fungal growth when the BM medium is used for the bacterial and fungal growth.

## SUPPLEMENTAL MATERIAL

Figure S1  Effect of DMDS on the growth of *A*. *fumigatus* (*Af*). Download Figure S1, PDF file, 0.4 MB

Figure S2  Schematic of the test setup and determination of VOC concentrations. Download Figure S2, PDF file, 0.7 MB
