# Supplementary material for: Volatile Compounds Emitted by Pseudomonas aeruginosa Stimulate Growth of the Fungal Pathogen Aspergillus fumigatus
Source: mBio. 2016 Mar 15;7(2):e00219-16. doi: 10.1128/mBio.00219-16 (PMC4807360; doi:10.1128/mBio.00219-16)
Supplement: Figure S1 — Effect of DMDS on the growth of A. fumigatus (Af). Download [file mbo002162728sf1.pdf]

**SI Figure 1**

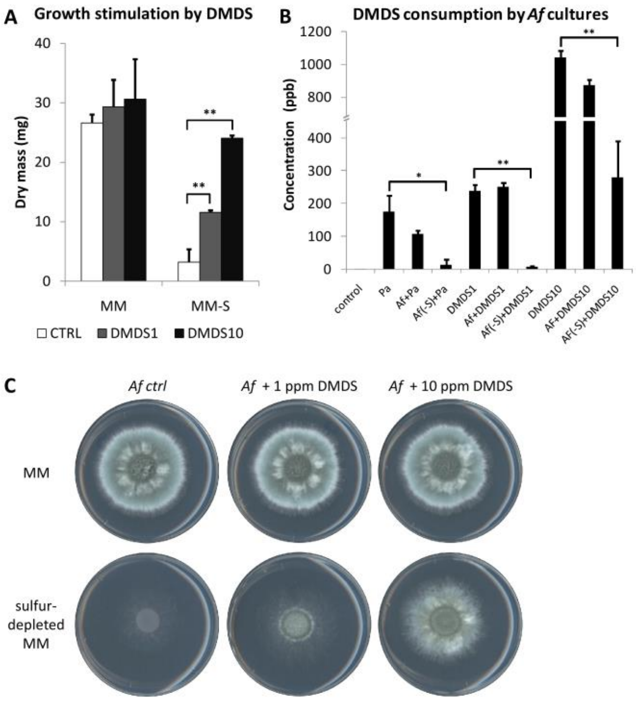


**SI Figure 1: Effect of DMDS on the growth of *Af*. (A)** DMDS at 1 or 10 ppm concentration stimulates the growth of *Af* on sulfur-depleted medium (MM-S) but not on sulfur replete medium (MM). **(B)** Residual concentration of DMDS in the culture headspace of the double plate setup. DMDS produced by *Pa* (grown on MM) or the pure compound (1 and 10 ppm) is not significantly taken up by the fungus when grown on MM (*Af*), but is consumed on sulfur-deficient MM (*Af*(-S)). **(C)** Growth of *Af* in the presence of DMDS in the culture headspace. Growth is not affected on MM, whereas growth is increased on sulfur-depleted MM in a dose-dependent manner, as observed for DMS (Fig. 2D). All error bars show the values' S.E.M.; $^{*}$ $p \leq 0.05$; $^{**}$ $p \leq 0.01$.
