# Supplementary material for: Volatile Compounds Emitted by Pseudomonas aeruginosa Stimulate Growth of the Fungal Pathogen Aspergillus fumigatus
Source: mBio. 2016 Mar 15;7(2):e00219-16. doi: 10.1128/mBio.00219-16 (PMC4807360; doi:10.1128/mBio.00219-16)
Supplement: Figure S2 — Schematic of the test setup and determination of VOC concentrations. Download [file mbo002162728sf2.pdf]

SI Figure 2

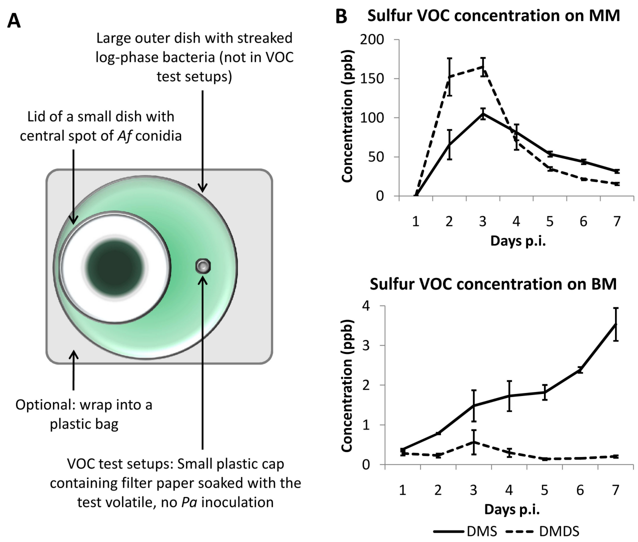

SI Figure 2: (A) Schematic of the test setup used in this work**. (B)** Determination of concentrations of DMS and DMDS produced by *Pa* in minimal medium (MM) and Brian's broth (BM). Days p.i.: days post inoculation. Error bars show the values' S.E.M.
